# Lipid changes after interleukin-6 blockade in rheumatoid arthritis: beyond cholesterol elevation toward hepatic inflammatory-lipoprotein remodeling

**DOI:** 10.3389/fcvm.2026.1847053

**Published:** 2026-06-23

**Authors:** Na Yuan, Zipei Zhang, Xiancheng Wang, Ting Li

**Affiliations:** 1 Department of Rheumatology, The Affiliated Hospital to Changchun University of Chinese Medicine, Changchun, China; 2 Graduate Student Administration Office, The Affiliated Hospital to Changchun University of Chinese Medicine, Changchun, China; 3 Cardiology Department, The Third Affiliated Hospital of Changchun University of Traditional Chinese Medicine, Changchun, China; 4 Senile Diseases Department, The Third Affiliated Hospital of Changchun University of Traditional Chinese Medicine, Changchun, China; 5 Department of Liver and Stomach Diseases, The Affiliated Hospital to Changchun University of Chinese Medicine, Changchun, China

**Keywords:** cardiovascular risk, hepatic lipid remodeling, interleukin-6 blockade, lipid paradox, rheumatoid arthritis

## Abstract

Rheumatoid arthritis is associated with excess cardiovascular morbidity and mortality despite the frequent presence of relatively low circulating lipid levels during active disease, a phenomenon commonly described as the lipid paradox. Interleukin-6 is central to this paradox because it links synovial inflammation with hepatic acute-phase responses, lipoprotein remodeling, thromboinflammatory pathways, and vascular injury. In this narrative review, we examine how IL-6 blockade reshapes the interpretation of lipid changes in rheumatoid arthritis and whether post-treatment cholesterol elevation should be viewed as isolated metabolic harm or as part of a broader process of hepatic inflammatory-lipoprotein remodeling. Mechanistic and translational evidence suggests, but does not yet prove, that active rheumatoid arthritis is characterized not only by reduced lipid concentrations, but also by dysfunctional high-density lipoprotein, oxidative lipoprotein modification, increased serum amyloid A loading, and vascular inflammation. After IL-6 pathway inhibition, particularly with tocilizumab and sarilumab, total cholesterol and low-density lipoprotein cholesterol often increase early; however, these changes may occur alongside reductions in C-reactive protein, serum amyloid A, lipoprotein(a), fibrinogen, and D-dimers, as well as selected improvements in lipoprotein function. Current hard cardiovascular outcome data generally support cardiovascular neutrality rather than a clear increase in major adverse cardiovascular events, although uncertainty remains for specific outcomes, vascular territories, and patient subgroups. Overall, lipid elevation after IL-6 blockade should prompt contextual cardiovascular risk refinement rather than reflexive interpretation as isolated cholesterol-mediated harm.

## 1 Introduction

Rheumatoid arthritis, although clinically recognized as a chronic inflammatory joint disease, is now understood as a systemic immune-mediated disorder with a substantial excess burden of cardiovascular morbidity and mortality. Meta-analyses of observational studies have shown that patients with RA have an approximately 50% higher risk of cardiovascular mortality and a 48% higher risk of incident cardiovascular events than the general population, with notable increases in myocardial infarction and stroke (1, 2). This excess risk is sufficiently consistent that contemporary EULAR recommendations incorporate cardiovascular risk assessment into routine RA management rather than treating it as a peripheral concern (3).

The explanation for this cardiovascular burden extends beyond traditional risk factors alone. RA-associated vascular disease is increasingly viewed as the product of persistent cytokine-driven inflammation, endothelial dysfunction, oxidative stress, immune activation, and inflammation-induced alterations in lipoprotein biology. In this setting, circulating lipids are not merely passive biomarkers of metabolic health; they become part of an inflammatory vascular network in which particle composition, oxidative susceptibility, and downstream thromboinflammatory signaling may matter as much as, or more than, the absolute cholesterol concentration itself (4).

This broader context helps explain why the so-called lipid paradox has emerged as one of the most intriguing observations in cardio-rheumatology. In the general population, lower total cholesterol and lower LDL-C are usually interpreted as favorable. In RA, however, lower lipid concentrations may coexist with higher inflammatory activity and greater cardiovascular risk. In a population-based cohort, Myasoedova and colleagues showed that systemic inflammation, particularly erythrocyte sedimentation rate, was strongly linked to cardiovascular outcomes, whereas lower total cholesterol and LDL-related measures did not provide the expected protection under inflammatory conditions (5). Imaging-based work subsequently reinforced this paradox by showing that RA patients with the lowest circulating LDL concentrations could nevertheless carry a greater burden of subclinical coronary atherosclerosis (6). Together, these observations indicate that in active inflammatory disease, low measured cholesterol may sometimes reflect inflammation-suppressed lipid levels rather than true vascular protection.

Among the inflammatory pathways implicated in RA, interleukin-6 is especially important because it sits at the intersection of synovial inflammation, hepatic acute-phase biology, lipoprotein remodeling, and vascular risk. IL-6 is not simply another pro-inflammatory cytokine. It is a major regulator of the hepatic acute-phase response and thereby influences a series of liver-derived proteins that connect inflammation to metabolism and atherothrombosis (7). This liver-centered perspective becomes particularly relevant when considering serum amyloid A and related acute-phase mediators, because inflammatory loading of SAA onto HDL can alter HDL composition and function in ways that are not captured by routine lipid measurements (8). As a result, once IL-6 signaling is blocked, changes in cholesterol concentrations may reflect not only altered lipid quantity but also a deeper resetting of hepatic inflammatory and lipoprotein biology.

For this reason, IL-6 blockade provides a useful clinical model for revisiting the lipid paradox in RA. Tocilizumab and other IL-6 pathway inhibitors reproducibly increase conventional lipid fractions, especially total cholesterol and LDL-C, raising concern about potential metabolic liability (9, 10). However, mechanistic and translational studies indicate that these changes should not automatically be equated with uniform worsening of cardiovascular biology. Strang and colleagues reported that tocilizumab-induced lipid changes were accompanied by reduced hepatic LDL receptor expression, suggesting that part of the lipid signal may arise from hepatic effects (9). The MEASURE trial showed that although IL-6 receptor blockade increased LDL-C, it also shifted HDL-associated inflammatory proteins and modified several vascular risk surrogates, including lipoprotein(a), fibrinogen, and D-dimers (10). A similar biomarker pattern has been reported with sarilumab, supporting—but not proving—the possibility of a class-relevant effect on inflammatory-lipoprotein remodeling (11).

Accordingly, the central question is no longer whether IL-6 blockade raises lipids, because it clearly does in many patients. The more important question is how these lipid elevations should be interpreted when inflammatory suppression simultaneously alters hepatic acute-phase signaling, lipoprotein composition, and thromboinflammatory tone (7–11). In this review, we examine whether lipid elevations after IL-6 blockade in RA may be better understood within a liver-centered inflammatory-lipoprotein framework, while explicitly distinguishing mechanistic and biomarker evidence from vascular surrogate findings and hard cardiovascular outcomes.

For this narrative review, we conducted a targeted literature search of PubMed/MEDLINE, Web of Science, and Google Scholar from database inception to March 15, 2026. Search terms included combinations of “rheumatoid arthritis”, “interleukin-6”, “tocilizumab”, “sarilumab”, “lipid paradox”, “cholesterol”, “LDL-C”, “HDL function”, “serum amyloid A”, “lipoprotein(a)”, “hepatic lipid metabolism”, “acute-phase response”, “cardiovascular risk”, “major adverse cardiovascular events”, “vascular inflammation”, and “statin”. This was a narrative rather than systematic review; therefore, no formal protocol registration, duplicate screening, or risk-of-bias scoring was performed. We prioritized randomized trials, large observational cohort studies, mechanistic human studies, vascular imaging studies, systematic reviews, and guideline-relevant documents. When evidence was conflicting, we weighted hard cardiovascular outcomes above vascular surrogate markers, and vascular surrogate or biomarker findings above mechanistic hypotheses.

## 2 The pre-treatment paradox: why active rheumatoid arthritis appears lipid-poor but remains atherogenic

Before the effects of IL-6 blockade can be interpreted correctly, it is necessary to understand the lipid milieu of untreated or inadequately controlled RA. Active RA is frequently associated with an abnormal lipoprotein pattern characterized not by overt hypercholesterolemia, but by relatively low concentrations of circulating total cholesterol, LDL-C, and, most consistently, HDL-C (12, 13). This pattern is not unique to RA and resembles the lipid suppression observed in other high-grade inflammatory states, suggesting that systemic inflammation itself can lower circulating lipid levels (13). Yet the clinical meaning of these low lipid concentrations differs sharply from that in the general population, because higher inflammatory activity in RA has been linked to greater cardiovascular risk, including myocardial infarction (14). Likewise, vascular imaging studies have shown that patients with RA may exhibit increased carotid intima-media thickness despite an otherwise favorable conventional risk profile, supporting the view that chronic inflammation can accelerate atherosclerosis even when traditional lipid metrics appear reassuring (15).

This apparent contradiction becomes more intelligible when lipid changes are examined alongside inflammatory trajectories rather than in isolation. In a longitudinal RA cohort, reduction in hs-CRP over time was accompanied by increases in LDL-C and apolipoprotein A1, but also by improvement in HDL cholesterol efflux capacity (16). These findings are highly informative because they indicate that falling inflammation can unmask higher circulating lipid levels while simultaneously improving a key antiatherogenic function of HDL. In other words, low lipid concentrations in active RA should not automatically be interpreted as metabolically favorable; they may instead reflect an inflammation-driven state in which lipid quantity is suppressed while lipid quality and vascular biology are deteriorating (13, 16).

The inadequacy of standard lipid measurements in RA becomes even clearer when HDL function is examined directly. HDL from patients with active RA has impaired capacity to promote cholesterol efflux, indicating compromised reverse cholesterol transport even when conventional HDL-C values alone do not fully capture this defect (17). More broadly, inflammation induces marked remodeling of the HDL proteome, with increases in serum amyloid A and reductions in apolipoprotein A-I, paraoxonase 1, and other proteins that normally support anti-oxidative and anti-inflammatory functions (18). Consistent with this concept, a triglyceride-high, HDL-low phenotype in RA has been associated with higher inflammatory cytokine levels, reduced PON1 activity, and poorer response to TNF blockade (19). Recent work has further shown that RA patients exhibit reduced HDL antioxidant capacity and lower resistance of LDL particles to oxidation, with these abnormalities relating largely to inflammatory burden rather than to lipid concentration alone (20).

These functional disturbances are not merely biochemical curiosities; they map onto vascular injury. In RA, autoantibodies against oxidized LDL correlate positively with CRP, inversely with HDL cholesterol, and independently with carotid intimal thickening, suggesting that inflammation-linked lipoprotein modification may contribute to subclinical atherosclerosis (21). Similarly, in a cross-sectional study of RA patients, higher HDL cholesterol efflux capacity was independently associated with a lower probability of carotid plaque, while low and moderate disease activity were associated with lower efflux capacity than remission (22). Taken together, these data support a model in which active RA promotes atherogenesis not simply through altered cholesterol levels, but through dysfunctional lipoproteins, impaired cholesterol handling, oxidative modification, and persistent arterial inflammation (17, 20–22).

From this perspective, the pre-treatment lipid paradox in RA is not truly a paradox of cholesterol quantity alone, but a mismatch between standard lipid readouts and the underlying biology of cardiovascular risk. As Choy and Sattar argued, in severe inflammatory states the reduction of inflammation may lead to higher lipid concentrations without necessarily increasing cardiovascular risk, because the rise in cholesterol may partly reflect reversal of inflammation-induced lipid suppression rather than worsening atherogenic exposure (23). This conceptual shift is essential for interpreting subsequent lipid changes during IL-6 blockade. If active RA is a state in which low measured lipids coexist with dysfunctional HDL, oxidatively vulnerable LDL, and accelerated arterial injury, then post-treatment increases in cholesterol cannot be judged adequately without considering the accompanying changes in inflammation, hepatic acute-phase signaling, and lipoprotein quality (16–23).

## 3 IL-6 at the liver–vessel interface: a mechanistic basis for the paradox

The mechanistic importance of IL-6 in RA extends beyond synovial inflammation. Through classic and trans-signaling pathways, IL-6 connects immune activation with hepatocyte acute-phase responses, endothelial activation, and vascular inflammation (24). Because hepatocytes are central targets of IL-6 signaling, the liver is particularly relevant when interpreting metabolic and lipoprotein changes after therapeutic IL-6 blockade.

This liver-centered role is fundamental to the acute-phase response. Human hepatocyte studies have shown that IL-6 directly induces acute-phase protein synthesis, including C-reactive protein, serum amyloid A, fibrinogen, and haptoglobin (25, 26). For lipoprotein biology, serum amyloid A is especially relevant because it can replace constitutive apolipoproteins on HDL and thereby contribute to inflammatory remodeling of HDL structure and function (27). IL-6-related haemostatic effects, including fibrinogen-linked pathways, further connect hepatic inflammatory signaling with atherothrombotic risk (28).

Other liver-derived IL-6-regulated mediators, such as hepcidin and haptoglobin, further illustrate that IL-6 blockade can reset a broader hepatic inflammatory program, although these pathways are less directly related to lipid interpretation (29, 30).

Lipoprotein(a) provides a more direct bridge between hepatic biology and vascular risk. Experimental work in primary monkey hepatocytes showed that IL-6 increased Lp(a) accumulation and apo(a) mRNA expression, suggesting cytokine-level regulation of this liver-derived atherogenic particle (31). Human data are directionally consistent with this mechanism: IL-6 blockade with tocilizumab reduced circulating Lp(a), linked hepatic IL-6-response genes with LPA expression, and inhibited IL-6-induced LPA mRNA and protein expression in human hepatocytes, whereas TNF-α inhibition did not produce the same effect (32). These findings are relevant to the lipid paradox because IL-6 blockade may reduce Lp(a) even when conventional LDL-C rises, but they should be interpreted as mechanistic and biomarker evidence rather than proof of lower clinical event risk (32).

**Figure 1:**
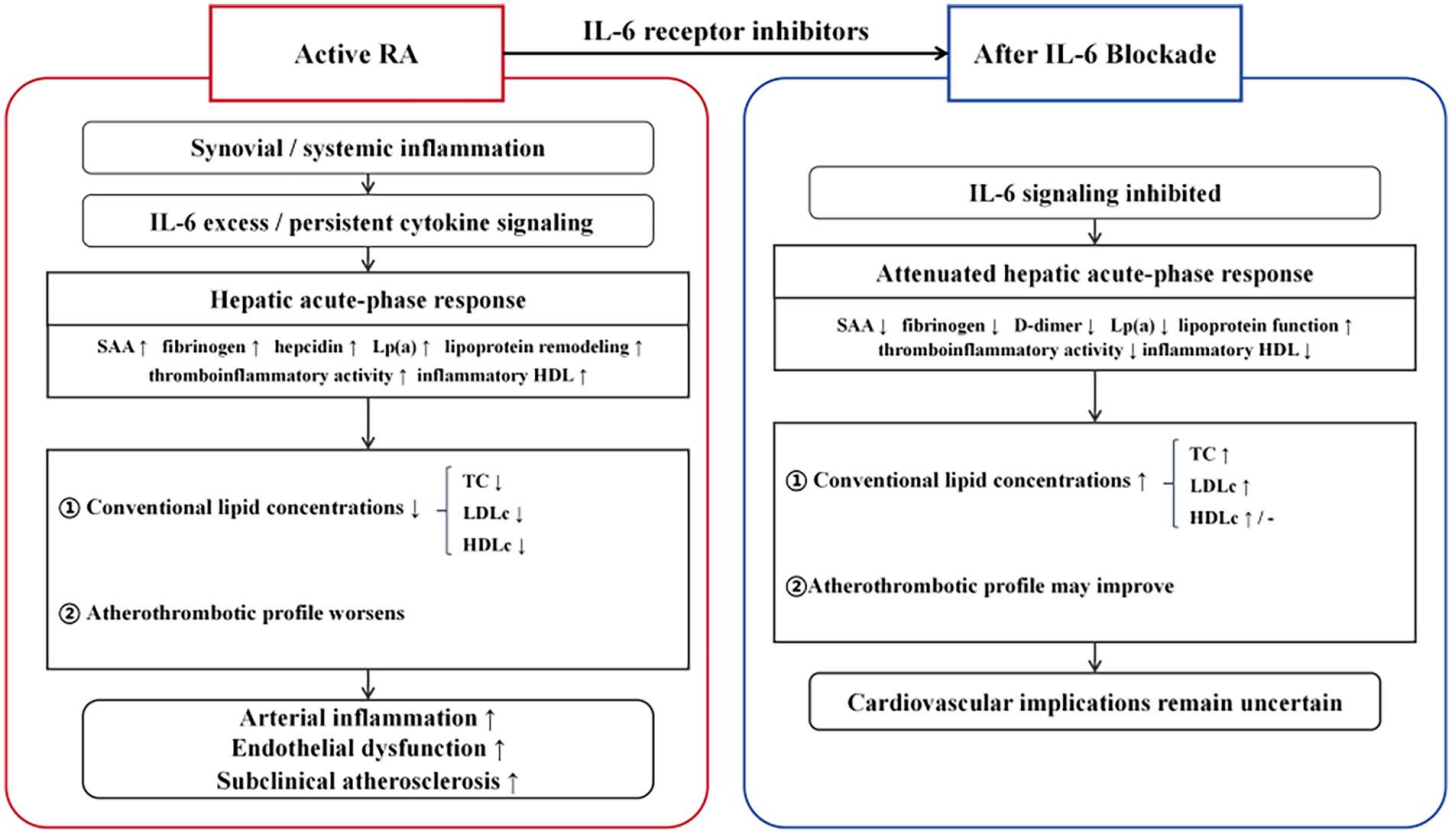
Liver–vessel framework linking the rheumatoid arthritis lipid paradox with IL-6 blockade-induced hepatic inflammatory-lipoprotein remodeling. In active rheumatoid arthritis, systemic inflammation and increased IL-6 signaling may suppress measured lipid concentrations, leading to relatively low total cholesterol, LDL-C, and HDL-C despite persistent vascular inflammation and increased cardiovascular risk. At the liver–vessel interface, IL-6 promotes hepatic acute-phase responses and contributes to serum amyloid A enrichment of HDL, lipoprotein (a) regulation, fibrinogen-related thromboinflammatory signaling, and qualitative lipoprotein remodeling. After IL-6 pathway inhibition, particularly with tocilizumab and sarilumab, conventional lipid fractions such as total cholesterol and LDL-C often increase, while selected inflammatory-lipoprotein and thromboinflammatory markers, including CRP, serum amyloid A, lipoprotein (a), fibrinogen, and D-dimers, may decrease. This framework is intended to integrate the pre-treatment RA lipid paradox with post-treatment hepatic inflammatory-lipoprotein remodeling, but it remains hypothesis-generating and should be interpreted separately from hard cardiovascular outcome evidence.

Beyond individual acute-phase proteins, IL-6 may also influence hepatic lipid handling through JNK/STAT- and ERK/MAPK-linked pathways, although these effects are context-dependent (33). In RA, translational studies after IL-6 receptor inhibition suggest that tocilizumab can remodel the protein cargo of VLDL, LDL, and HDL, reduce proinflammatory proteins associated with HDL particles, preserve selected HDL functions, and reduce oxidized LDL or Lp(a) in some cohorts (34–36). These observations support a plausible liver–vessel framework in which conventional lipid fractions may rise while selected inflammatory, lipoprotein-functional, and thromboinflammatory signals improve. However, this remains a mechanistic interpretation, and the net cardiovascular consequence must be judged against vascular surrogate and hard-outcome data. A conceptual summary of this proposed liver–vessel framework is shown in Figure 1.

## 4 What changes after IL-6 blockade? Evidence for hepatic lipid remodeling

The clinical lipid signal of IL-6 blockade is one of the most reproducible metabolic observations in RA therapeutics. In a systematic review and meta-analysis of randomized clinical trials, tocilizumab, but not TNF antagonists, was associated with moderate increases in total cholesterol, LDL-C, and HDL-C, confirming that this pattern is not anecdotal but a recurring pharmacologic effect across trial settings (37). This quantitative shift is already evident in the MEASURE trial, in which median total cholesterol, LDL-C, and triglyceride levels rose by week 12 in tocilizumab-treated patients compared with placebo, while total HDL-C did not change significantly between groups (10). Importantly, the biologic interpretation of this pattern becomes more nuanced once particle composition and liver-linked inflammatory markers are examined alongside conventional lipid fractions (10).

The most informative feature of the post-IL-6 lipid phenotype is that quantity and quality do not move in parallel. In MEASURE, despite the increase in LDL-C, there were no significant differences in small LDL or oxidized LDL concentrations between groups, while HDL-associated serum amyloid A decreased and paraoxonase increased in patients receiving tocilizumab (10). At the same time, lipoprotein(a), fibrinogen, D-dimers, and secretory phospholipase A2-IIA were all reduced by more than 30%, and the ApoB/ApoA1 ratio remained stable over time (10). These changes are difficult to reconcile with a purely simplistic interpretation of IL-6 blockade as merely proatherogenic. Instead, they suggest that IL-6 inhibition can raise measured cholesterol concentrations while simultaneously attenuating several liver-linked acute-phase and atherothrombotic signals that may also contribute to vascular risk.

This pattern is not limited to tocilizumab alone. In the MONARCH biomarker analysis, sarilumab monotherapy produced substantially greater reductions than adalimumab in CRP, serum amyloid A, and lipoprotein(a) by week 24, with the reduction in Lp(a) reaching 41.0% with sarilumab vs. 2.8% with adalimumab (11). Because SAA and Lp(a) are closely linked to hepatic inflammatory output, these findings are consistent with the possibility that IL-6 pathway inhibition has a class-relevant effect on liver-centered inflammatory-lipoprotein remodeling rather than an isolated effect on cholesterol concentration alone (11). However, the most detailed lipid-composition and vascular-surrogate data are available for tocilizumab, whereas sarilumab provides supportive but more limited evidence for a broader IL-6 pathway effect. By contrast, TNF inhibitors, abatacept, rituximab, and JAK inhibitors may influence cardiovascular risk through partly different inflammatory, lipid, and safety profiles, so the lipid paradox observed after IL-6 blockade should not be generalized uncritically to all targeted RA therapies.

The temporal profile of these changes is also clinically important. In a longitudinal study of RA patients receiving long-term tocilizumab, total cholesterol rose earliest at 12 weeks, whereas non-HDL cholesterol, LDL-C, HDL-C, and triglycerides peaked at 24 weeks, then moved back toward baseline by week 52; notably, total cholesterol/HDL-C and LDL-C/HDL-C ratios, as well as the atherogenic index of plasma, did not change significantly over time (38). Lipid changes were inversely correlated with changes in DAS28 and CDAI, again suggesting that part of the apparent dyslipidemia reflects the resolution of inflammatory suppression rather than straightforward worsening of atherogenic burden (38). The rapidity of this phenomenon was further illustrated in the ENTRACTE trial, where LDL-C, HDL-C, and triglycerides were already significantly higher by week 4 in patients receiving tocilizumab than in those receiving etanercept (39). Taken together, these data indicate that IL-6 blockade induces an early and clinically visible rise in circulating lipid fractions, but also suggest that the broader lipid phenotype evolves in parallel with inflammatory control and may not be adequately interpreted through cholesterol concentration alone (10, 38, 39).

A final practical issue is whether these biochemical changes are modifiable in routine care. In a pooled *post hoc* analysis of seven phase 3 and 4 studies, concomitant statin use attenuated tocilizumab-mediated lipid increases over 2 years without a clinically significant increase in adverse events, although lipid-lowering therapy remained underused among eligible patients (40). These findings indicate that the post-IL-6 lipid signal is clinically manageable, even if its biological interpretation remains more complex than a simple rise in cholesterol concentration alone (40).

## 5 From hepatic remodeling to vascular biology: how should we interpret the signal?

The key translational question is whether post-treatment biochemical changes after IL-6 blockade are accompanied by measurable changes in vascular surrogate markers. Small prospective studies suggest that this may occur for selected vascular readouts. In a pilot study of active RA, six monthly infusions of tocilizumab were associated with progressive improvement in flow-mediated dilation and reduction in pulse wave velocity (41). In an open-label randomized trial comparing tocilizumab, etanercept, and adalimumab monotherapy, all three strategies reduced arterial stiffness over 24 weeks, but only tocilizumab increased total cholesterol, while none changed carotid intima-media thickness or carotid plaque burden (42). A 12-month observational study likewise found that tocilizumab increased total cholesterol, LDL-C, and HDL-C without changing the LDL-C/HDL-C ratio or carotid intima-media thickness (43).

At the same time, these surrogate vascular data should not be overinterpreted as proof of rapid cardiovascular benefit. In a *post hoc* analysis of a randomized trial, NT-proBNP decreased similarly in both placebo and tocilizumab arms over 24 weeks, whereas hsTnT also fell in both groups but to a lesser extent in the tocilizumab arm, leading the investigators to conclude that IL-6 receptor blockade did not show a rapid preferential benefit on these specific cardiac biomarkers (44). This is an important cautionary signal, because it indicates that different surrogate readouts may move in different directions and on different timescales after IL-6 inhibition. Vascular benefit, if present, is therefore unlikely to be captured adequately by any single biomarker or by short-term assessment alone.

Thromboinflammatory markers appear to respond more consistently to IL-6 blockade. In an observational study of RA patients treated with tocilizumab, prothrombin fragment F1 + 2 and D-dimer levels fell significantly after four weeks, in parallel with reductions in DAS28, ESR, CRP, and TNF-α (45). These findings are directionally concordant with the MEASURE trial, in which fibrinogen and D-dimers were also reduced after IL-6 receptor inhibition (10). Broader vascular physiology may also improve, although the pattern is selective rather than uniform. In a randomized study comparing anakinra, tocilizumab, and prednisolone, tocilizumab improved pulse wave velocity and brachial blood pressure, whereas anakinra showed greater effects on myocardial deformation and coronary flow reserve (46). At the evidence-synthesis level, a systematic review concluded that tocilizumab and other non-TNF biologics were associated with favorable effects on endothelial dysfunction, whereas effects on arterial stiffness were less consistent and no significant effect was demonstrated for measures of subclinical atherosclerosis (47). Taken together, surrogate vascular data complicate a simple cholesterol-centered interpretation of post-IL-6 lipid elevation, but they do not by themselves establish clinical benefit.

An alternative explanation should also be acknowledged. The rise in conventional lipid fractions after IL-6 blockade may partly reflect reversal of inflammation-driven lipid suppression rather than a distinct and fully characterized hepatic remodeling program (5, 13, 16, 23). In addition, improvement in selected biomarkers or vascular surrogates does not necessarily translate into long-term cardiovascular benefit (39, 44, 47–52). It therefore remains possible that, in some patients or vascular territories, conventional lipid elevation still carries clinically relevant consequences that are not fully captured by currently available studies (39, 48–53). These observations should also be placed within the broader principle that effective suppression of systemic inflammation is itself relevant to cardiovascular risk management in RA. Recent RA data suggest that biological DMARD use may attenuate the association between inflammatory activity and major adverse cardiovascular events, and the TARGET trial showed that both TNF inhibition and triple therapy produced clinically meaningful reductions in arterial inflammation (54, 55). Evidence from outside RA further supports the concept that inflammatory pathway inhibition can reduce cardiovascular events independently of lipid lowering: in the CANTOS trial, greater hsCRP reduction after IL-1β inhibition with canakinumab was associated with greater cardiovascular event reduction without a lipid-lowering effect (56). Therefore, IL-6 blockade should be viewed not as an isolated lipid phenomenon, but as one example within a wider inflammation-targeted cardiovascular paradigm; however, this broader concept should not be taken as proof that IL-6 blockade itself reduces hard cardiovascular events in all RA populations (57).

## 6 Hard cardiovascular outcomes: vascular benefit, neutrality, or unresolved risk?

Hard cardiovascular outcomes provide the most clinically relevant, although still incomplete, test of the post-IL-6 lipid paradox. The strongest randomized evidence comes from ENTRACTE, in which the hazard ratio for major adverse cardiovascular events with tocilizumab vs. etanercept was 1.05, and the upper bound of the confidence interval ruled out a relative risk increase of 1.43 or greater (39). In practical terms, this trial did not show an excess of overall MACE with tocilizumab.

Real-world comparative effectiveness studies have generally pointed in the same direction. In a large multi-database population-based cohort of RA patients who switched from another biologic or tofacitinib, there was no evidence that tocilizumab increased composite cardiovascular risk compared with TNF inhibitors (48). In a subsequent multi-database cohort analysis, tocilizumab was associated with cardiovascular risk comparable to etanercept and also to abatacept, rituximab, and infliximab, further supporting the absence of a clear overall cardiovascular hazard signal (49). A separate multi-database comparison specifically evaluating tocilizumab vs. abatacept likewise found no significant difference in the risk of composite cardiovascular events across Medicare, PharMetrics, and MarketScan data (50). Together, these studies suggest that, at the level of broad composite cardiovascular outcomes, tocilizumab has most often been associated with cardiovascular neutrality rather than with an obvious excess-risk signal.

This interpretation is supported, though not definitively settled, by evidence synthesis. A 2024 systematic review and network meta-analysis of randomized clinical trials comparing JAK inhibitors or tocilizumab with TNF inhibitors did not identify a significant increase in MACE or all-cause death attributable to tocilizumab in RA (51). These findings suggest that the discordance between worsening conventional lipid fractions and non-worsening hard cardiovascular outcomes is reproducible beyond isolated cohorts.

At the same time, the story is not completely closed. In 2025, a Korean nationwide nested case-control study reported that current exposure to tocilizumab was associated with an increased risk of incident ischemic stroke, with an adjusted odds ratio of 3.47, although the authors explicitly cautioned that this finding should be interpreted carefully because of statistical limitations (52). This signal matters because it raises the possibility that different vascular beds may not respond identically to IL-6 blockade, and that composite cardiovascular endpoints may conceal heterogeneity between coronary disease, cerebrovascular disease, and possibly heart failure (52). It also underscores a broader methodological issue: observational associations with individual outcomes may be especially vulnerable to channeling bias, sparse exposure counts, time-varying confounding, and differences in baseline inflammatory severity (48–50, 52).

Overall, current outcome data support cardiovascular neutrality at the level of composite endpoints rather than clear cardioprotection or clear excess risk. Thus, the available hard-outcome evidence is compatible with a cautious reinterpretation of lipid elevation after IL-6 blockade, but it does not prove that hepatic inflammatory-lipoprotein remodeling translates into clinical cardiovascular benefit. At the same time, uncertainty remains for specific outcomes such as ischemic stroke and for subgroup- or phenotype-specific risk that may be obscured within broad composite cardiovascular endpoints (39, 48–52).

## 7 Clinical implications: a liver-centered Reading of post-IL-6 lipid changes

From a clinical standpoint, an important interpretive challenge is that post-IL-6 lipid elevations cannot be read in exactly the same way as lipid changes in a non-inflammatory population. In RA, conventional cardiovascular screening tools and traditional risk factors often underestimate vascular risk, and the lipid profile by itself is insufficient to capture the full cardiovascular signal in an inflammatory setting (3, 53). This does not mean that LDL-C elevations after tocilizumab should be ignored. Rather, it means that they should be read in context: alongside disease activity suppression, hepatic acute-phase resetting, changes in lipoprotein quality, and the broader vascular phenotype of the individual patient (10, 32, 36, 53).

This contextual approach is especially important because LDL-C does not directly quantify the number of circulating atherogenic particles. ApoB reflects the total number of apoB-containing lipoproteins and may therefore provide a more accurate estimate of residual atherogenic burden when cholesterol content per particle is variable (58). This concept is particularly relevant after IL-6 blockade, where conventional cholesterol fractions may rise even as inflammatory remodeling of lipoproteins shifts in a potentially less atherogenic direction (10, 34–36). For similar reasons, lipoprotein(a) deserves attention as a marker of residual cardiovascular risk, because elevated Lp(a) is independently associated with atherosclerotic cardiovascular disease and calcific aortic valve disease even when other lipid parameters appear acceptable (59). In the specific context of IL-6 inhibition, this is clinically appealing because tocilizumab has repeatedly been shown to reduce Lp(a), implying that part of the post-treatment lipid phenotype may actually represent mitigation of a high-risk liver-derived lipoprotein signal rather than a uniform worsening of cardiometabolic risk (10, 32, 36).

Non-HDL-C may also be useful in day-to-day practice because it captures cholesterol carried by all atherogenic apoB-containing particles rather than LDL alone. In a 2023 cohort of RA patients treated with biologics or JAK inhibitors, tocilizumab and JAK inhibitors were associated with greater increases in non-HDL-C than TNF inhibitors or abatacept, and these changes were not explained by improvement in DAS28, indicating that the non-HDL signal is not simply a mirror of declining inflammatory activity (60). Statin evidence in RA should also be considered. In TRACE RA, the largest double-blind randomized placebo-controlled statin trial in RA, atorvastatin 40 mg daily was safe and reduced LDL-C, although the trial was stopped early because of a lower-than-expected event rate and was therefore underpowered for cardiovascular events (61). This supports lipid-lowering therapy according to overall cardiovascular risk, while avoiding an automatic assumption that every IL-6-related lipid rise requires discontinuation of effective anti-inflammatory treatment (40, 60, 61).

A pragmatic clinical approach is to avoid both reflexive alarm and reflexive reassurance. Patients starting IL-6 blockade should undergo baseline cardiovascular risk assessment, including conventional lipid testing, and lipid reassessment should be performed early after treatment initiation because meaningful increases can appear within 4 weeks and often evolve through the first 12–24 weeks (3, 38, 39). When LDL-C or non-HDL-C rises materially, clinicians should optimize lipid-lowering therapy according to the patient's overall cardiovascular risk rather than discontinuing an otherwise effective anti-inflammatory treatment solely because total cholesterol has increased (40, 53, 60). In selected patients, especially those with metabolic syndrome, high triglycerides, discordance between LDL-C and the apparent clinical risk profile, or established atherosclerotic disease, additional measurement of ApoB and Lp(a) may improve interpretation of residual risk beyond the standard lipid panel alone (32, 54, 59).

## 8 Unresolved questions and future directions

Several key questions remain unresolved. First, cardiovascular risk prediction in RA remains inadequate. In early RA, four widely used cardiovascular risk algorithms showed poor performance for future cardiovascular events, and even RA-adapted SCORE algorithms did not provide sufficient improvement (62, 63). A recent review similarly emphasized that RA cardiovascular risk assessment requires attention to inflammatory burden, traditional risk factors, imaging, and individualized preventive strategies rather than reliance on a single general-population calculator (64). This limitation is directly relevant to IL-6 blockade, because treatment-related lipid changes are difficult to interpret when the baseline cardiovascular risk model is itself imprecise.

Second, future studies should move beyond standard lipid panels toward integrated vascular and biomarker phenotyping. Carotid ultrasound can improve cardiovascular risk stratification in RA, particularly by reclassifying patients initially considered to be at low or moderate risk by conventional algorithms (65). In parallel, biomarker studies such as TARGET suggest that inflammatory, metabolic, and cardiac-related markers may help characterize arterial inflammation and treatment-associated vascular changes (66). For IL-6 blockade specifically, future work should test whether ApoB, lipoprotein(a), HDL function, serum amyloid A-related measures, coagulation markers, vascular imaging, and other lipid-related residual-risk biomarkers outperform standard cholesterol fractions in predicting clinical outcomes after treatment (10, 32, 65, 66, 67).

Third, treatment effects should be studied in a phenotype-specific and outcome-specific way. Current studies usually report average lipid or cardiovascular effects across heterogeneous RA populations, but the consequences of IL-6 blockade may differ according to baseline inflammatory burden, obesity, insulin resistance, pre-existing atherosclerotic disease, and concomitant statin use (39, 40, 60, 68). Future studies should avoid relying solely on composite MACE definitions and should evaluate myocardial infarction, ischemic stroke, heart failure, vascular death, and peripheral vascular outcomes separately, especially in light of the emerging stroke-specific signal reported in recent observational work (52). Prospective cohorts combining serial lipid measurements, ApoB and Lp(a), HDL functional assays, inflammatory and coagulation markers, vascular imaging, and adjudicated cardiovascular events will be needed to determine whether post-IL-6 lipid elevation is mainly a biochemical epiphenomenon of inflammatory resolution, a marker of residual risk requiring more aggressive prevention, or both (10, 39, 48–52).

## 9 Conclusion

The IL-6 blockade lipid paradox in rheumatoid arthritis is best viewed not as a resolved contradiction, but as a hypothesis-generating reminder that post-treatment lipid changes cannot be interpreted through a conventional cholesterol-centered lens alone. In RA, IL-6 contributes not only to synovial inflammation but also to a broader liver-centered acute-phase and lipoprotein program involving serum amyloid A, lipoprotein(a), coagulation-related proteins, and qualitative properties of circulating lipoproteins. Once this pathway is inhibited, LDL-C and total cholesterol often rise, while selected inflammatory, thromboinflammatory, and lipoprotein-related signals may improve. Current evidence therefore supports cautious reinterpretation rather than dismissal of post-IL-6 lipid elevations. However, whether hepatic inflammatory-lipoprotein remodeling fully explains long-term cardiovascular risk remains unproven, and phenotype-specific risks, including cerebrovascular outcomes, require further study.
